# Hypothyroid Myopathy—A Rare Case from Paediatric Practice

**DOI:** 10.3390/children11040400

**Published:** 2024-03-28

**Authors:** Stanimira Elkina, Ventsislava Stoyanova, Irina Halvadzhiyan, Chayka Petrova

**Affiliations:** Department of Pediatrics, Medical University-Pleven, 5100 Pleven, Bulgaria; vency_slava@abv.bg (V.S.); irina.halvadjian@gmail.com (I.H.); petrovachayka@yahoo.com (C.P.)

**Keywords:** hypothyroidism, myopathy, Kocher–Debré–Semelaigne syndrome

## Abstract

Hypothyroid myopathy is uncommon in childhood. Severe hypothyroid myopathy observed in paediatric practice is a part of Kocher–Debré–Semelaigne syndrome (KDSS, OR-PHA:2349), a rare disorder characterised by muscular pseudohypertrophy and long-standing moderate-to-severe hypothyroidism. We present a pubertal girl with KDSS diagnosed with severe myopathy and significantly limited mobility and progressively increasing pains in the lumbar area, hip joints, and the lower limbs. Additionally, the patient presented metabolic syndrome with severe obesity, growth retardation, and educational difficulties. In this case, adequate hormone replacement therapy with Levothyroxine evoked full recovery of the myopathy and a significant reversal in the patient’s general condition. In conclusion, emphasizing the knowledge related to KDSS can improve the diagnosis and prognosis of the condition.

## 1. Introduction

Primary hypothyroidism with impaired function of the thyroid gland is one of the most common endocrine pathologies in childhood. On average, it affects about 5.3% (1.7–9.5%) of the paediatric population worldwide. Depending on its aetiology, primary hypothyroidism can be congenital or acquired. The main reason for the onset of acquired primary hypothyroidism described in the literature is the autoimmune destruction of the thyroid gland [1].

Autoimmune hypothyroidism (Hashimoto’s autoimmune thyroiditis) may manifest with a number of symptoms, such as hypertrophy of the gland due to goitre, weight gain, cold intolerance, dry skin, alopecia, deterioration of school performance, and growth delay. Rarely, the diagnosis of Hashimoto’s thyroiditis may be delayed. In cases of late diagnosis, the disease manifests mostly with atypical symptoms associated with different pubertal disorders (secondary amenorrhea or metrorrhagias), anaemia and coagulopathies, rarely pituitary enlargement, and various musculoskeletal manifestations [2,3]. One such atypical manifestation is hypothyroid myopathy. Hypothyroid myopathy may occur at any age. In adults, it is known as Hoffmann’s syndrome [4] and has a prevalence of less than 10%. In childhood, the condition is known as the Kocher–Debré–Semelaigne syndrome (KDSS), a rare disorder (ORPHA: 2349) characterised by muscular pseudohypertrophy due to long-standing moderate-to-severe congenital or acquired hypothyroidism [5]. The syndrome was initially described by Emil Kocher in 1892 but was conclusively defined in 1935 by Robert Debré and George Semelaigne [6]. The exact prevalence of KDSS and its distribution among genders is debatable [5]. Only single cases or a small series of cases has been described in the existing literature worldwide, while no comparable case has been published in the accessible Bulgarian literature.

We present a clinical case of severe hypothyroid myopathy caused by the late diagnosis of acquired autoimmune hypothyroidism. The patient and the family provided written informed consent prior to publication.

## 2. Case Description

A 13-year-old female was referred to the Paediatric Clinic of Dr. Georgi Stranski University Hospital, Pleven, for an evaluation of severe obesity. She was delivered naturally at term after an uncomplicated pregnancy with birth weight of 3.9 kg and birth length of 51 cm. Routine local national newborn screening was conducted—which, in Bulgaria, presently includes screening for congenital hypothyroidism, congenital adrenal hyperplasia, and phenylketonuria—and showed no evidence of congenital diseases. Normal early physical and psychomotor development were evident. Familial history of second-degree relatives with autoimmune diseases of the thyroid gland and a father with a metabolic syndrome (abdominal obesity, hypertension, and insulin resistance) was reported.

Overweight since an early-school age with a significant increase in weight over the last year and an improper diet, involving the daily consumption of large amounts of carbohydrates and fat-rich foods, were reported. She reported menarche at the age of 11, with secondary amenorrhea for the last 3 months. Her school performance had worsened over the past year. For the last couple of months, she had been suffering from severely limited mobility due to progressively increasing pains in the lumbar area, hip joints, and lower limbs. Due to the pain symptoms, the girl was referred for consultation to a paediatric neurologist in an outpatient setting, who referred her to a paediatric endocrinologist for obesity assessment.

**Figure 1:**
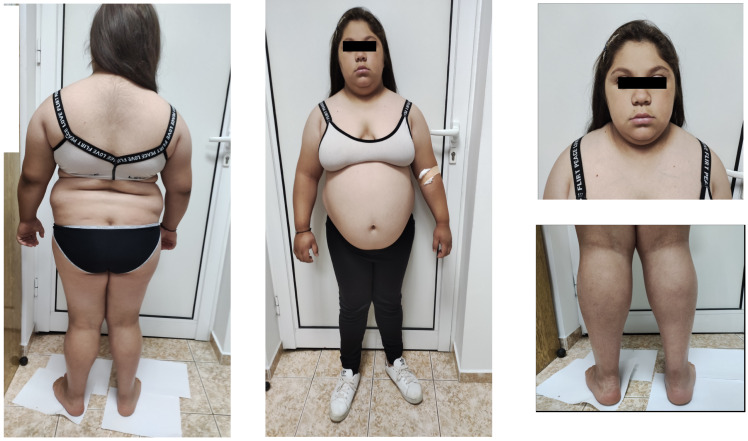
Patient’s physical appearance at diagnosis—short stature, abdominal obesity, coarse face, and muscle hypertrophy of bilateral calves.

At admission, the girl was in an impaired general condition. A “mask-like face” with rough facial features, thick lips, and macroglossia was notable on physical examination. She had very dry skin with acanthosis nigricans on the neck, axillae, and arms. Abnormal fatty tissue distribution with increased accumulation in the abdominal area was observed. No hyperplasia of the thyroid gland and no abnormalities in the respiratory and cardiovascular systems were evident. She was in the fifth Tanner stage of pubertal development. The patient experienced difficulties in rising from a lying or sitting position, inability to get up from a crouching position, and severe difficulty climbing up and down stairs. Pain on palpation bilateral around the spine at the lumbar vertebrae level was present. Lasègue’s sign and Patrick’s tests were bilaterally positive. Significant symmetrical pseudohypertrophy of the lower legs, flat feet, and pathological walk were noted. She had decreased knee and Achilles reflexes with preserved sensitivity. The patient responded slightly slower than normal during the interview and the whole physical examination. The anthropometric measurements showed evidence of short stature and severe obesity: a height of 141 cm (−2.83 SDS), a weight of 77 kg (+1.91 SDS), a BMI of 38.7 kg/m^2^ (+2.88 SDS), and a waist circumference of 110 cm (Figure 1).

The laboratory tests showed elevated levels of liver enzymes, cholesterol, and triglycerides, as well as substantially increased creatine kinase. Furthermore, insulin resistance with impaired glucose tolerance and without diabetic levels of blood glucose during oral glucose tolerance test was determined. The laboratory results and the clinical presentation evidenced the presence of metabolic syndrome, with our patient fulfilling three of all four criteria for its diagnosis (abdominal obesity, dyslipidaemia with elevated triglycerides and reduced HDL, and impaired glucose tolerance [7]). The hormonal evaluation evidenced severe autoimmune hypothyroidism (Table 1).

**Figure 2:**
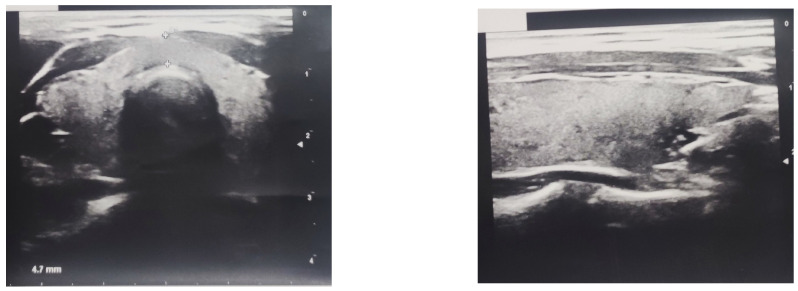
Ultrasound scan of patient’s hypoechogenic, non-homogeneous-structured, normal-sized thyroid gland.

The ultrasound scan showed a hypoechogenic, non-homogeneous-structured, and normal-sized thyroid gland (Figure 2), as well as normal structures and sizes of the liver and the spleen.

Due to the clinical complaints related to the musculoskeletal system, an electromyography (EMG) and a CT scan of the lumbosacral spine and the hip joints were performed. The EMG showed evidence of myogenic impairment of the lower limbs, while the CT scan showed no evidence of anatomical impairments. Due to the combination of extremely elevated muscle enzymes with pseudohypertrophy of the lower legs presented in our patient, Duchenne muscular dystrophy (DMD) was suspected. To exclude DMD, genetic counselling and microarray-based comparative genomic hybridisation (mCGH array) testing were performed. No deletions and duplications in dystrophin gene were found, and congenital muscular dystrophy was ruled out.

**Figure 3:**
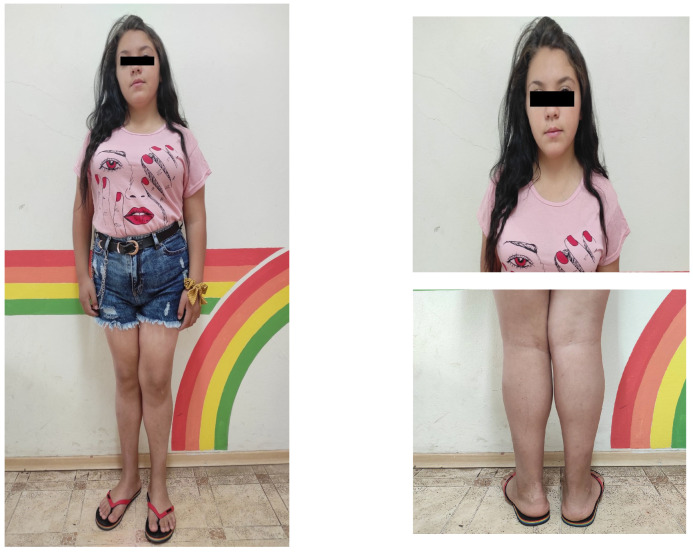
Patient’s physical appearance after one-year hormonal treatment—reduced weight and resolved muscle hypertrophy of bilateral calves.

Based on the thyroid gland pathology and the clinical manifestation in our patient, she was diagnosed with Kocher–Debré–Semelaigne syndrome. Replacement therapy with L-thyroxine (L-T4) at an adjustable dosage was initiated. Our patient was administered an initial dose of 1 mcg/kg of L-T4 after her cortisol levels were found to be within the normal range with testing. The L-T4 dose was slowly built up gradually to the current dose of 2.5 mcg/kg. As an initial treatment for metabolic syndrome, lifestyle changes involving a hypocaloric diet and adequate physical activity were recommended. A nutritional education of the patient’s family was performed. The girl was put on a moderately low energy diet with a daily caloric target of not more than 1200 kcal. An increased intake of whole grains, fresh fruits, and vegetables was recommended, along with eating regular meals four times daily with controlled portions, avoiding snacking and limiting fried and battered foods. She was encouraged to increase her physical activity stepwise to 60 min daily and to limit screen time to maximum 2 h daily. The patient was trained to fill in a daily food diary. On every follow-up visit, the data from the food diary were analysed, and a nutritional re-education was performed. The follow-up of the patient showed very good results from the conducted therapy, but, due to metabolic abnormalities with persisting impairments to glucose tolerance and high levels of basal insulin, Metformin (1000 mg/day) was added to the therapy. One year after the start of the treatment, the girl presented with the complete reversal of the muscular pseudohypertrophy of the lower legs and a significant reduction in body weight, with a height of 148 cm (−2.13 SDS), a weight of 61 kg (+0.81 SDS), a BMI of 27.8 kg/m^2^ (+1.62 SDS), and a waist circumference of 86 cm (−24 cm in 12 months), see Figure 3. On the latest follow-up (March 2024), her TSH and FT4 levels were within normal ranges, and the metabolic markers were fully normal. Finally, the patient presented improved motor function and a substantial improvement in her school performance against the background of continuing titration of the hormone replacement therapy.

## 3. Discussion

The clinical manifestations of hypothyroidism also include symptoms involving the musculoskeletal system, such as muscle hypotonia or isolated pains in the muscles and the joints [4,8]; however, severe myopathies are not common in children. KDSS is a rare hypothyroid myopathy that is developed in childhood, characterised by the muscular pseudohypertrophy of the lower limbs. Short stature with a typical “Heracles-like” appearance and a neuropsychological development delay may also be observed. The KDSS is not widely known, and only a small series of cases or single cases are reported sporadically in the literature. Therefore, the diagnosis of this condition is delayed or could be even missed. It is most often diagnosed between the ages of 18 months and 10 years, but there are also reports of cases diagnosed during the neonatal period [6,9,10]. The majority of the reported KDSS cases are at an early age due to the presence of congenital hypothyroidism. In these cases, the hypothyroid myopathy is mostly combined with short stature and/or global mental delay [11,12]. Panat et al. [6] and Patnay et al. [13] presented patients with KDSS associated with orofacial pathologies. Congenital hypothyroidism affects bone maturation and teeth development and eruption, causing impaired dentition and jaw deformities. In last decades congenital hypothyroidism is timely diagnosed due to newborn screening programs; therefore, KDDS due to congenital hypothyroidism is uncommon [10,11]. There are some recent reports of KDSS due to acquired hypothyroidism. Kankananarachchi et al. [10] described a boy with consanguineous parents exhibiting ichthyosis and KDSS due to acquired autoimmune hypothyroidism. The prevalence and gender distribution of KDSS are debatable: while some authors have reported that it has no gender prevalence, there are also data in the literature indicating a higher prevalence of the syndrome among boys born from consanguineous marriages [6,10,13]. Our patient is diagnosed with KDSS at a later age than usually reported for this syndrome. She is a pubertal girl born from a non-consanguineous marriage with KDSS due to severe Hashimoto’s autoimmune thyroiditis. During diagnosis, she presented with a short stature, but no orofacial malformations or skin diseases were notable. She had acanthosis nigricans due to insulin resistance and secondary amenorrhea as a result of severe obesity and hyperandrogenism. She showed some educational problems, but no severe global mental delay was diagnosed.

In the medical literature, the negative impact of chronic diseases, including hypothyroidism, on a patient’s quality of life is widely suggested [14]. At the time of diagnosis, our patient presented with an overall impaired quality of life. She showed impaired cognitive and physical abilities. Her school performance worsened gradually below average, and she experienced difficulties in memorizing new information. She reduced her interaction with her schoolmates. Because of severe myopathy, our patient’s daily physical activity was limited. While on treatment, the girl reported a better self-esteem, with predominant positive emotions. Her school grades and communication with peers are improved, and she is more physically active.

The severity of the myopathy generally correlates to the duration and severity of the thyroid hormone deficiency [6]. The pathogenesis of the muscular pseudohypertrophy in cases of KDSS has not yet been fully explained. Thyroid-stimulating hormone (TSH) and the thyroid hormones (FT4 and FT3) play major roles in a number of metabolic functions in humans. It has been proven that the impaired carbohydrate metabolism in cases of long-standing hypothyroidism leads to the accumulation of glycogen in muscles, while the increased accumulation of connective tissue and mucopolysaccharides in the striated muscles contributes to muscle hypertrophy [15,16].

The thyroid hormones regulate the metabolism of calcium ions in the cells and influence the activity of the calcium-dependent adenosine triphosphate (ATP). This decrease in ATP activity slows the muscle relaxation speed, leading to a decrease in calcium return to the sarcoplasmic reticulum [17]. The associated slowing of muscle contractions and relaxations are the causes of the generalised slowing of movement. In most cases, the electromyogram is normal; however, it may show evidence of myogenic impairment [13], which was observed in the case of our patient.

On the other hand, the thyroid hormones participate in the regulation of the expression of genes involved in the syntheses and functions of the fast myofibrillar proteins (type II myofibrils) in the striated muscles. In the event of evident thyroid hormone deficiency, a change occurs in the muscle fibres that involves an increase in the composition of slow myofibrils (type I) [14].

As a result of the changes described above, the elevated levels of creatine kinase (CK) are often observed in patients with hypothyroidism. Furthermore, in patients with untreated hypothyroidism, the energy production during physical exertion is insufficient, as a result of which rhabdomyolysis with an extreme increase in CK is seen [15].

The differential diagnosis of hypothyroid myopathy involves a wide range of inflammatory, metabolic, congenital, and acquired neuromuscular diseases, such as polymyositis, myasthenia gravis, myelomeningocele, and amyotrophic lateral muscular sclerosis. Due to the increased levels of muscle enzymes and muscular pseudohypertrophy, a primary muscle disorder must be ruled out. Duchenne muscular dystrophy (DMD) is a primary muscle disorder inherited in a recessive X-linked manner. It is diagnosed usually in boys, but there are rare cases of female patients due to the inactivation of the chromosome carrying the non-mutated copy of the gene [18]. In cases of unclear diagnoses, a muscle biopsy is recommended. In KDSS, such a biopsy would provide evidence of the accumulation of glycogen or necrosis in the interstitial tissue [16]. In the case of our patient, imaging tests (CT scan) ruled out the anatomical impairment of the spine or the hip joints, while a genetic test eliminated the carriage of the gene for DMD. It was decided that performing a muscle biopsy would not be necessary.

It is known that hypothyroid myopathy in cases of KDSS is reversible with an adequate hormone replacement therapy [16]. After the initiation of Levothyroxine treatment, our patient experienced a significant improvement in muscle power, with the complete reversal of the pseudohypertrophy of the lower legs. A significant reduction in body weight and the normalisation of metabolic abnormalities were also observed.

The relationship between the functioning of the thyroid gland and the manifestation of metabolic syndrome symptoms is complex. The thyroid hormones participate—directly or indirectly—in the metabolism of all tissues in an organism. According to some studies, there is a positive association between the increased level of TSH, in cases of hypothyroidism, and increased body weight and triglyceride levels. On the other hand, the reduced thyroid hormone levels reduce the utilisation of blood glucose, causing a pathological elevation of its levels [7]. Moreover, in cases of hypothyroidism, pathologically elevated levels of liver enzymes are observed; again, a positive correlation between their levels and the TSH level has been described [19]. It is thought that the increases in ALT and GGT levels are due to the worsened lipid metabolism and hepatic steatosis, which occur in cases of hypothyroidism, while the elevated levels of AST and LDH are related to hypothyroid myopathy [20]. Like in cases of hypothyroid myopathy, the prompt start of a hormone replacement therapy may lead to improvements in metabolic markers and weight reduction, as was evident in the case of our patient.

## 4. Conclusions

Primary autoimmune hypothyroidism may manifest at any time during childhood, with a number of typical or rare symptoms, such as hypothyroid myopathy, and is known as KDSS. An adequate hormone replacement therapy in cases of KDSS leads to the reversal of the disease symptoms and the full recovery of the affected muscle groups. Due to the increased levels of muscle enzymes and muscular pseudohypertrophy, it is crucial to distinguish KDSS from a primary muscle disorder. In future actively describing more cases in detail could be useful for enhancing the knowledge of hypothyroid myopathy in physicians’ daily practice. Comprehending the Kocher–Debré–Semelaigne syndrome and intentionally considering it when examining children with a decreased function of the thyroid gland can improve the prognosis of the disease and increase the quality of life for patients.

## Figures and Tables

**Table 1:** Laboratory tests at diagnosis, after one-year hormonal treatment, and on latest follow-up.

Parameter		At Diagnosis	After 1 Year of Treatment	Latest Follow-Up	Reference Range
Haemoglobin	g/L	121	130	127	120–145
ASAT	U/L	121.87	18.5	24.0	<35
ALAT	U/L	133.55	16.0	19.5	<49
Alkaline phosphatase	U/L	63.39	140.0	120	35–140
GGT	U/L	83.24	11.0	16.0	<40
Creatine kinase	U/L	2665.54	63.0	98.8	<170
Creatine kinase–MB	U/L	57.2	21	15.0	<24
Cholesterol	mmol/L	7.83	3.35	4.2	<5.2
Triglycerides	mmol/L	12.86	0.55	1.13	<2.3
HDL	mmol/L	0.82	1.2	1.49	>1.5
OGTT–0′ glucose	mmol/L	4.96	4.64	5.1	<5.6
OGTT–120′ glucose	mmol/L	8.74	7.6	7.5	<7.8
Basal insulin	mL/UmL	16.0	11.0	10.3	4.0–16.0
HOMA-IR		3.5	2.3	2.3	<2.5
TSH	mIu/L	143.0	7.24	3.9	0.2–4.0
FT4	pg/mL	1.7	11.6	14.3	7.0–17.8
Anti TPO	UI/L	1200	600	120	<60
Anti TG	UI/L	69	40	45	<60
FSH	UI/L	4.1	3.9	3.8	0.3–9.0
LH	UI/L	6.9	4.5	5.1	0.1–10.6
Oestradiol	pg/mL	120	135	126	35–147
Testosterone	nmol/L	4.8	1.6	1.9	0–3.9

## Data Availability

Data are contained within the article.
